# Interventional nephrology: current status and clinical impact in Japan

**DOI:** 10.1007/s10157-017-1457-y

**Published:** 2017-08-02

**Authors:** Masato Ikeda, Hiroyuki Terawaki, Eiichiro Kanda, Maiko Furuya, Yudo Tanno, Masatsugu Nakao, Yukio Maruyama, Masutaka Maeda, Chieko Higuchi, Tsutomu Sakurada, Tomohiro Kaneko, Hiroaki Io, Koji Hashimoto, Atsushi Ueda, Keita Hirano, Naoki Washida, Hiraku Yoshida, Kazuhiro Yoshikawa, Yoshihiro Taniyama, Kenji Harada, Nanae Matsuo, Ichiro Okido, Takashi Yokoo

**Affiliations:** 1 Division of Nephrology and Hypertension, The Jikei University School of Medicine Katsushika Medical Center, 6-41-2 Aoto, Katsushika-ku, Tokyo 125-8506 Japan; 2 Dialysis Center, Fukushima Medical University Hospital, 1 Hikariga-oka, Fukushima, 960-1295 Japan; 3 Department of Nephrology, Tokyo Kyosai Hospital, 2-3-8 Nakameguro, Meguro-ku, Tokyo, 153-8934 Japan; 4 Division of Nephrology and Hypertension, Department of Internal Medicine, Jikei University School of Medicine, 3-25-8 Nishi-shinbashi, Minato-ku, Tokyo 105-8461 Japan; 5 Nephrology Division, Department of Internal Medicine, JA Toride Medical Center, 2-1-1 Hongo, Toride, Ibaraki 302-0022 Japan; 6 Division of Internal Medicine, Tokyo Women’s Medical University Medical Center East, 1-10-2 Nishiogu, Arakawa-ku, Tokyo 116-8567 Japan; 7 Division of Nephrology and Hypertension, Integrated Care Center for Kidney Disease, St. Marianna University School of Medicine, 2-16-1 Sugao, Miyamae-ku, Kawasaki, Kanagawa 216- 8511 Japan; 8 Division of Nephrology, Department of Internal Medicine, Nippon Medical School, 1-1-5 Sendagi, Bunkyo-ku, Tokyo, 113-8603 Japan; 9 Division of Nephrology, Department of Internal Medicine, Juntendo University Faculty of Medicine, 2-1-1 Hongo, Bunkyo-ku, Tokyo, 113-8421 Japan; 10 Department of Nephrology, Shinshu University School of Medicine, 3-1-1 Asahi, Matsumoto, Nagano 390-8621 Japan; 11 Tsukuba University Hospital Hitachi Medical Education and Research Center, Jonan-cho 2-1-1, Hitachi, Ibaraki 317-0077 Japan; 12 Division of Nephrology, Department of Internal Medicine, Ashikaga Red Cross Hospital, 284-1 Yobe-cho, Ashikaga, Tochigi 326-0843 Japan; 13 Department of Endocrinology, Metabolism and Nephrology, Keio University School of Medicine, 35 Shinanomachi, Shinjyuku-ku, Tokyo 160-8582 Japan; 14 Hiraku Clinic, 5-18-9 Kamisoshigaya, Setagaya-ku, Tokyo 157-0065 Japan; 15 Department of Nephrology, Iwate Prefectural Central Hospital, 1-4-1 Ueda, Morioka-shi, Iwate 020-0066 Japan; 16 Department of Nephrology, Kinki University School of Medicine, 377-2 Ohno-higashi, Osakasayama-shi, Osaka 589-8511 Japan; 17 Division of Nephrology, Kokura Memorial Hospital, 3-2-1 Asano, Kokurakita-ku, Kitakyushu-shi, Fukuoka 802-8555 Japan

**Keywords:** Nephrologist, Peritoneal, Dialysis, Vascular access, Endovascular, Kidney biopsy, Intervention

## Abstract

**Background:**

Current status and clinical significance of interventional nephrology has not been reported from Japan.

**Methods:**

Questionnaires were mailed twice to the directors of all 534 Japanese certificated nephrology training institutions in 2014. The main questions were current performance, categorized annual procedure volume and managers of peritoneal dialysis (PD) access, vascular access (VA) surgery, endovascular intervention, and kidney biopsy. Frequencies of nephrologist involvement between high volume center and low volume center and association between the level of nephrologists’ involvement to each procedure and annual procedure volume were examined.

**Results:**

332 (62.2%) institutions answered performance of all procedures and 328 (61.4%) institutions answered all procedure volume. Kidney biopsy, VA surgery, endovascular intervention and PD access surgery were performed by any doctors in 94.2, 96.3, 88.4, and 76.2% and each involvement of nephrologist was 93.9, 54.1, 53.1 and 47.6%, respectively. Cochran–Armitage analyses demonstrated significant increases in all 4 procedure volume with greater management by nephrologists (*p* < 0.01). Nephrologists involvement to VA surgery associated with procedure volume increase in not only VA surgery, but also PD catheter insertion (*p* < 0.01) and kidney biopsy (*p* < 0.05). And nephrologists involvement to PD catheter insertion also associated with surgical volume increase in both VA surgery (*p* < 0.01) and endovascular intervention (*p* < 0.05).

**Conclusions:**

Main manager of all 4 procedures was nephrologist in Japan. Each procedure volume increased as nephrologists become more involved. Acquisition of one specific procedure by nephrologist associated with increase not only in this specific procedure volume, but also the other procedure volume.

## Introduction

Most fellows in non-nephrology internal medicine subspecialties have never considered nephrology as a career choice and the lack of procedural opportunities may be a major reason for not selecting nephrology as a career choice [1–3]. “Don’t nephrologists perform any procedures?” medical students sometimes ask nephrologists not only in the United States [4], but also in Japan. In fact, nephrologists are managing ordinary dialysis therapy, though various non-nephrologists are performing procedures related to nephrology around the world. This fragmentation does not optimize medical care and may be inconvenient to the patient.

In 2000, the American Society of Diagnostic and Interventional Nephrology (ASDIN) was established to change the views of nephrologists regarding the practice of nephrology by promoting procedural aspects [4, 5]. Responding these actions, several countries have reported on the current status of interventional nephrology [6, 7], but the exact proportions of procedures related to nephrology currently performed by nephrologists remains unclear [8]. Which doctors manage procedures related to nephrology and how many cases are performed in Japanese nephrology training institutions are unknown. Which specialties have positive effects on increasing case numbers are also unknown. If a certain procedure is performed by nephrologists, whether this has positive effects on the other procedure volume is likewise unclear.

No meetings comparable to ASDIN have been organized in Japan. We therefore, established the Japanese Meeting for Interventional Nephrology in 2013, and conducted the first questionnaire survey of all 534 Japanese adult nephrology training institutions to clarify the actual conditions of interventions related to nephrology. This questionnaire asked what types and how many procedures were performed by what medical specialties in each hospital.

The results of this survey firstly demonstrated variability between institutions in the performance of procedures related to nephrology by various specialists in Japan, and revealed main managers of this field are nephrologists in Japan and procedure volume increases with increasing levels of involvement by nephrologists.

## Materials and methods

### Methods

The survey was written jointly by the authors based on their experience in clinical nephrology, performance and assessment of skills in nephrology-related procedures. The general distribution of case numbers for each procedure underwent preliminary field testing with 15 authors, all of whom were members of Japanese nephrology training institutions. Details of this survey were decided by the Japanese Meeting for Interventional Nephrology in July 2013 and February 2014. To improve response rates, we selected a minimal number of procedures for the present questionnaire and excluded placement of hemodialysis catheters from the present survey. The ethics committee for clinical research at Jikei University School of Medicine approved all protocols in this study [Permission no. 26-003 (7508)].

Certification in procedures for nephrology fellows in Japan requires that training in these skills be obtained in a nephrology training institution. We thus chose Japanese certificated adult nephrology training institutions as target institutions for this study. Questionnaires were mailed to the directors of all 534 Japanese nephrology training institutions at that time in July 2014. A follow-up questionnaire was posted again in November 2014. All responses were collected by fax.

The questionnaire comprised 4 sections, with questions about access procedures for peritoneal dialysis (PD) (PD catheters insertion, removal, and unroofing) [9, 10]; hemodialysis vascular access (VA) procedures (VA surgery and endovascular intervention) and kidney biopsy (Table 1).

**Table 1:**
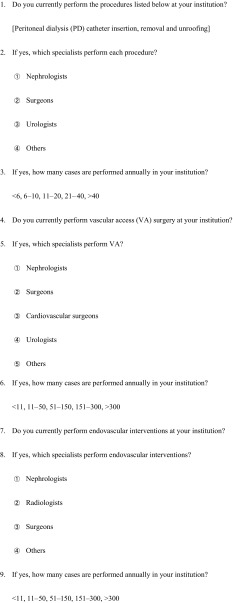

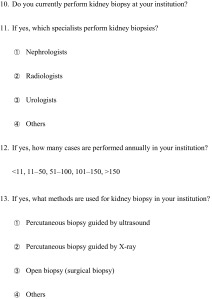
Questionnaire used in the present survey

### Data analysis

Staff of the Japanese Meeting for Interventional Nephrology reviewed the questionnaires and developed the final analysis.

### Institutional groupings

We divided institutions into 3 groups: N-institutions, with only nephrologists perform a specific procedure; non-N-institutions, with only non-nephrologists performing the specific procedure; and C-institutions, with both nephrologists and non-nephrologists cooperatively performing the specific procedure.

### Statistics

Statistical analyses were performed using JMP version 9.0 (SAS Institute, Cary, NC, USA). Data are expressed as mean ± standard deviation or numbers and percentages of institutions. Comparisons across groups were performed using the Pearson’s Chi-square test for categorical data. All tests were two-tailed, and values of *p* < 0.05 were considered significant. Cochran–Armitage analysis was used to compare case numbers for procedures among the 3 groups of N-institutions, non-N-institutions and C-institutions.

## Results

### Responding rate

Of the 534 Japanese certificated nephrology training institutions contacted, 236 (44.2%) institutions responded to the initial letters in July 2014, and additional 96 (18.0%) institutions responded to the follow-up letter in November 2014, resulting in responses from a total of 332 (62.2%) institutions. We examined differences of responding rate among each Japanese region and found minimum responding rate was 54.6% (18/33) in Chugoku region without significant differences. Though significant difference of responding rate was seen only in Hokkaido region [Hokkaido region 86.7% (13/15) vs the other regions 61.5% (319/519), *p* < 0.05], there was no significant differences among procedure performance, occupancy rates of nephrologists and procedure volume between Hokkaido region and the other regions.

### Distribution of annual procedure volume

Table 2 shows the distribution of annual procedure volume of key procedures. 328 institutions which were performing all 4 procedures in their own institutions by any type of doctors were included in this analysis. Annual case numbers of PD catheter insertions were categorized, and numbers and frequencies were as follows: <6 cases, 65.2% (*n* = 214; total responses: 328); 6–10 cases, 20.4% (*n* = 67); 11–20 cases, 11.3% (*n* = 37); 21–40 cases, 2.1% (*n* = 7); and >40 cases, 0.9% (*n* = 3). The majority of institutions (65.2%) performed <6 cases annually by any doctors.

**Table 2:** Distribution of annual cases of key procedures

VA surgery						
Annual cases	<11	11–50	51–150	151–300	>300	Total
Number of institutions	31	136	134	20	7	328
Frequency (%)	9.5%	41.5%	40.9%	6.1%	2.1%	100.0%
Endovascular intervention						
Annual cases	<11	11–50	51–150	151–300	>300	Total
Number of institutions	90	120	71	32	15	328
Frequency (%)	27.4%	36.6%	21.6%	9.8%	4.6%	100.0%
Kidney biopsy						
Annual cases	<11	11–50	51–100	101–150	>150	Total
Number of institutions	64	179	58	24	3	328
Frequency (%)	19.5%	54.6%	17.7%	7.3%	0.9%	100.0%
PD catheter insertion						
Annual cases	<6	6–10	11–20	21–40	>40	Total
Number of institutions	214	67	37	7	3	328
Frequency (%)	65.2%	20.4%	11.3%	2.1%	0.9%	100.0%
PD catheter removal						
Annual cases	<6	6–10	11–20	21–40	>40	Total
Number of institutions	285	37	4	1	1	328
Frequency (%)	86.9%	11.3%	1.2%	0.3%	0.3%	100.0%
PD catheter unroofing						
Annual cases	<6	6–10	11–20	21–40	>40	Total
Number of institutions	325	3	0	0	0	328
Frequency (%)	99.1%	0.9%	0.0%	0.0%	0.0%	100.0%

328 institutions which performed all 4 procedures in their own institutions were included in this analysis. Annual procedure volume of VA surgery, endovascular interventions, kidney biopsies, PD catheter insertions, removal and unroofing were categorized, and each number and frequencies were presented

*VA* vascular access, *PD* peritoneal dialysis

**Table 3:** Frequencies of procedure performance and involvement to procedure by nephrologists, non-nephrologists or their collaboration

	PD catheter insertion	PD catheter removal	PD catheter unroofing	Vascular access surgery	Endovascular intervention	Kidney biopsy
Total performing institutions	250 (76.2)	250 (76.2)	250 (76.2)	316 (96.3)	290 (88.4)	309 (94.2)
Nephrologistsª	75 (30.0)	72 (28.8)	89 (35.6)	110 (34.8)	113 (39.0)	263 (85.1)
collaborationª	44 (17.6)	38 (15.2)	26 (10.4)	61 (19.3)	41 (14.1)	27 (8.7)
Non-nephrologistsª	131 (52.4)	140 (56.0)	135 (54.0)	145 (45.8)	136 (46.9)	19 (6.1)

328 institutions answered all 4 procedures volume were included in this analysis. Table 3 shows frequencies of procedure performance and involvement to procedure by nephrologists, non-nephrologists or their collaboration. Each performance rates by any doctors were 96.3% for VA surgery (316/328 institutions), 94.2% for kidney biopsy (309/328 institutions), 88.4% for endovascular interventions (290/328 institutions) and 76.2% for PD access procedures (250/328 institutions)

Among 250 institutions which were offering PD access surgery by any doctors, frequencies of involvement to each PD access surgery by nephrologists, non-nephrologists or their collaboration were as follows: for PD catheter insertion, 30.0% (*n* = 75) vs 52.4% (*n* = 131) vs 17.6% (*n* = 44); for PD catheter removal, 28.8% (*n* = 72) vs 56.0% (*n* = 140) vs 15.2% (*n* = 38); for PD catheter unroofing, 35.6% (*n* = 89) vs 54.0% (*n* = 135) vs 10.4% (*n* = 26). Among non-nephrologists, surgeons (24.0%, *n* = 60/250) and urologists (24.4%, *n* = 61/250) were involved in higher degree of PD catheter insertions

**Fig. 1:**
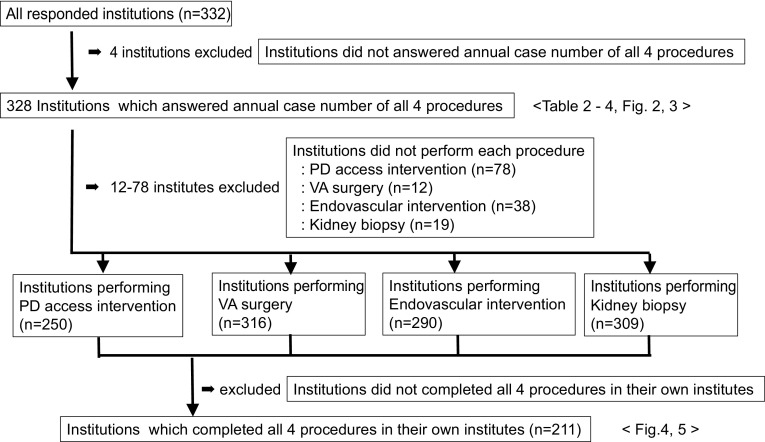
Institutions were selected and included in each analysis. Each inclusion criteria and included institution number for indicated Figures and Tables are showing

Frequencies of involvement by nephrologists, non-nephrologists and their collaboration in VA surgery, endovascular interventions and kidney biopsies were as follows: for VA surgery, 34.8% (*n* = 110) vs 45.8% (*n* = 145) vs 19.3% (*n* = 61; total responses: 316); for endovascular interventions, 39.0% (*n* = 113) vs 46.9% (*n* = 136) vs 14.1% (*n* = 41; total responses: 290); and for kidney biopsy, 85.1% (*n* = 263) vs 6.1% (*n* = 19) vs 8.7% (*n* = 27; total responses: 309) (Fig. 1)

^a^Values represent number (%) of institutions in which indicated doctors were offering indicated procedure among each procedure performing institutions

Annual case numbers of PD catheter removal were categorized, and numbers and frequencies were as follows: <6 cases, 86.9% (*n* = 285; total responses: 328); 6–10 cases, 11.3% (*n* = 37); 11–20 cases, 1.2% (*n* = 4); 21–40 cases, 0.3% (*n* = 1); and >40 cases, 0.3% (*n* = 1). The majority of institutions (86.9%) performed <6 cases annually by any doctors.

Annual case numbers of PD catheter unroofing were categorized, and numbers and frequencies were as follows: <6 cases, 99.1% (*n* = 325; total responses: 328); 6–10 cases, 0.9% (*n* = 3); and >10 cases, 0% (*n* = 0). The majority of institutions (99.1%) performed <6 cases annually by any doctors.

Annual case numbers of VA surgery were categorized, and numbers and frequencies were as follows: <11 cases, 9.5% (*n* = 31; total responses: 328); 11–50 cases, 41.5% (*n* = 136); 51–150 cases, 40.9% (*n* = 134); 151–300 cases, 6.1% (*n* = 20); and >300 cases, 2.1% (*n* = 7). The majority of institutions (82.4%) performed VA surgery 11–150 cases per year by any doctors.

Annual numbers of endovascular interventions were categorized, and numbers and frequencies were as follows: <11 cases, 27.4% (*n* = 90; total responses: 328); 11–50 cases, 36.6% (*n* = 120); 51–150 cases, 21.6% (*n* = 71); 151–300 cases, 9.8% (*n* = 32); and >300 cases, 4.6% (*n* = 15). The majority of institutions (64.0%) performed endovascular interventions <51 cases annually by any doctors.

Annual numbers of kidney biopsies were categorized, and number and frequencies were as follows: <11 cases, 19.5% (*n* = 64; total responses: 328); 11–50 cases, 54.6% (*n* = 179); 51–100 cases, 17.7% (*n* = 58); 101–150 cases, 7.3% (*n* = 24); and >150 cases, 0.9% (*n* = 3). Over half of institutions (54.6%) performed 11–50 kidney biopsies annually by any doctors.

### Frequencies of procedure performance and involvement to procedure by nephrologists, non-nephrologists or their collaboration

328 institutions answered all 4 procedures volume and were included in this analysis. Table 2 shows frequencies of procedure performance and involvement to procedure by nephrologists, non-nephrologists or their collaboration. Each performance rates by any doctors were 96.3% for VA surgery (316/328 institutions), 94.2% for kidney biopsy (309/328 institutions), 88.4% for endovascular interventions (290/328 institutions), and 76.2% for PD access procedures (250/328 institutions).

### Comparison of sharing rates of procedure performance by nephrologists, 1 non-nephrologists, and both acting cooperatively

Among 250 institutions which were offering PD access surgery by any doctors, frequencies of involvement to each PD access surgery by nephrologists, non-nephrologists or their collaboration were as follows: for PD catheter insertion, 30.0% (*n* = 75) vs 52.4% (*n* = 131) vs 17.6% (*n* = 44); for PD catheter removal, 28.8% (*n* = 72) vs 56.0% (*n* = 140) vs 15.2% (*n* = 38); for PD catheter unroofing, 35.6% (*n* = 89) vs 54.0% (*n* = 135) vs 10.4% (*n* = 26). Among non-nephrologists, surgeons (24.0%, *n* = 60/250) and urologists (24.4%, *n* = 61/250) were involved in higher degree of PD catheter insertions.

Frequencies of involvement by nephrologists, non-nephrologists and their collaboration in VA surgery, endovascular interventions and kidney biopsies were as follows: for VA surgery, 34.8% (*n* = 110) vs 45.8% (*n* = 145) vs 19.3% (*n* = 61; total responses: 316); for endovascular interventions, 39.0% (*n* = 113) vs 46.9% (*n* = 136) vs 14.1% (*n* = 41; total responses: 290); and for kidney biopsy, 85.1% (*n* = 263) vs 6.1% (*n* = 19) vs 8.7% (*n* = 27; total responses: 309) (Fig. 1).

Among non-nephrologists, cardiac surgeons (19.0%, *n* = 60/316), urologists (13.0%, *n* = 41/316), surgeons (8.9%, *n* = 28/316) and others (5.1%, *n* = 16/316) performed VA surgery. Radiologists (23.1%, *n* = 67/290), cardiac surgeons (11.4%, *n* = 33/290), cardiologists (7.6%, *n* = 22/290), surgeons (1.7%, *n* = 5/290) and others (3.1%, *n* = 9/290) performed endovascular interventions. Urologists (4.9%, *n* = 15/309) and others (1.3%, *n* = 4/309) performed kidney biopsies.

68.9% (226/328) institutions could complete all 4 procedures in their own institutions by any doctors. And 22.9% (75/328) institutions could complete all 4 procedures by nephrologists (including nephrologists in cooperative action with non-nephrologists). The resultant 46.0% (151/328) institutions needed other specialists to complete all 4 procedures.

### Methods of native kidney biopsy

In this survey, we also investigated method of kidney biopsy in Japanese certificated adult nephrology training institutions. Kidney biopsy (total responded institutions: 309) was performed by ultrasound (US)-guided in 99.0% (*n* = 306). 92.2% (*n* = 285) used US-guided procedures alone, 6.1% (*n* = 19) used both US-guided or open biopsy, and 0.6% (*n* = 2) used either US- or X-ray-guided procedures. The remaining institutions used open biopsy alone (0.6%, *n* = 2) or some other method details unknown (0.3%, *n* = 1). There were no institutions performing X-ray-guided kidney biopsy alone.

### Differences of nephrologist involvement levels between high and low volume center

**Fig. 2:**
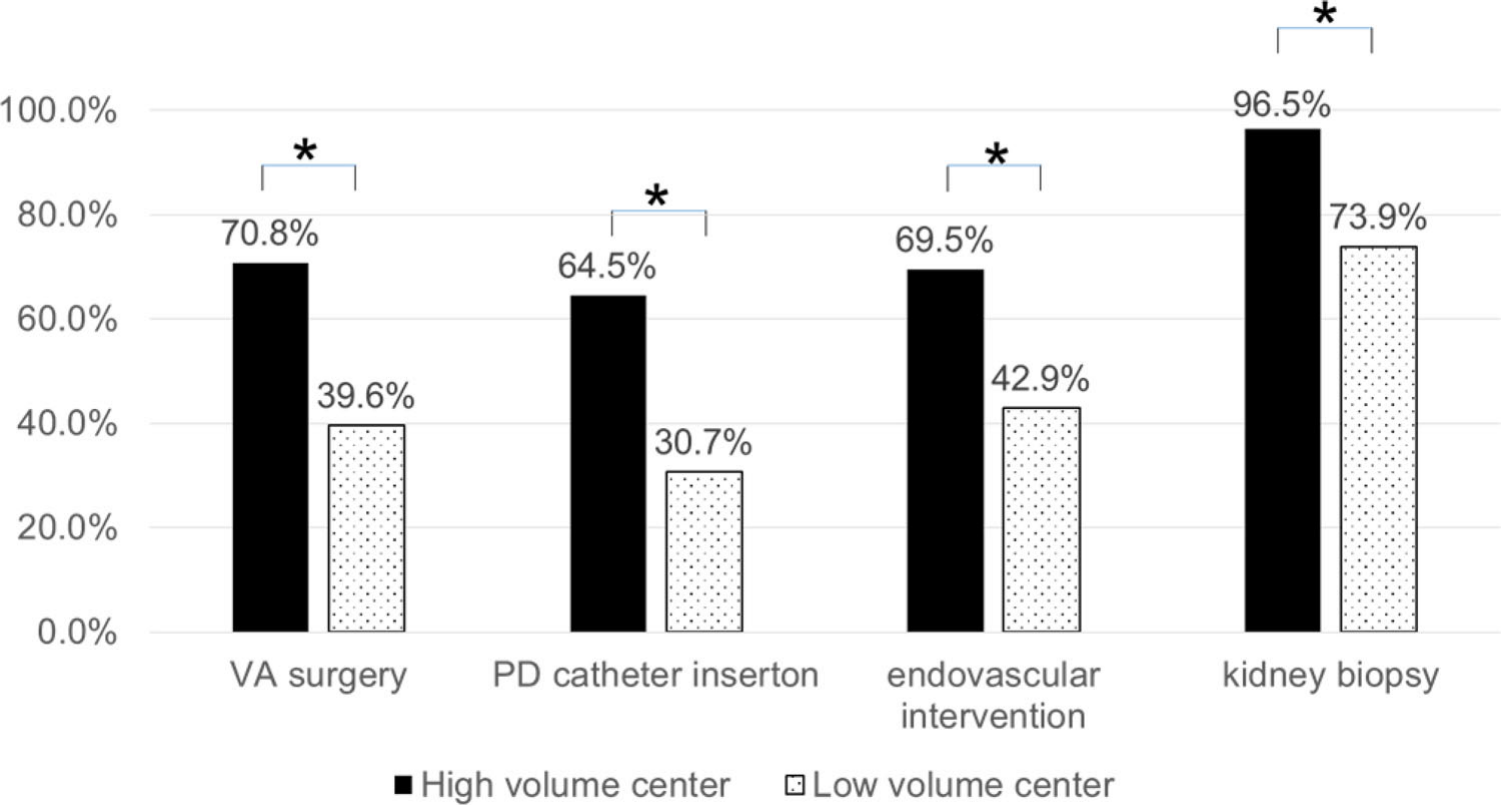
Differences of nephrologist involvement levels between high and low volume center. Frequencies of nephrologist involvement between high and low volume center were 70.8% (92/130) and 39.6% (36/91), *p* < 0.0001 for >50 cases and not more than 50 cases year of VA surgery, 64.5% (69/107) and 30.7% (35/114), *p* < 0.0001 for >5 cases and no more than 5 cases per year of PD catheter insertion, 69.5% (66/95) and 42.9% (54/126), *p* < 0.0001 for >50 cases and not more than 50 cases per year of endovascular intervention, 96.5% (191/198) and 73.9% (17/23), *p* < 0.0001 for >10 cases and not more than 10 cases per year of kidney biopsy, respectively. Values represent the percentage of nephrologist involvement. *p < 0.01

To clarify association between frequencies of nephrologist involvement to each intervention and procedure volume, we compared the frequencies of nephrologist involvement between high volume center and low volume center in each 4 procedure (Fig. 2). Frequencies of nephrologist involvement between high and low volume center were 70.8% (92/130) and 39.6% (36/91), *p* < 0.01 for >50 cases and no more than 50 cases year of VA surgery, 64.5% (69/107) and 30.7% (35/114), *p* < 0.01 for >5 cases and no more than 5 cases per year of PD catheter insertion, 69.5% (66/95) and 42.9% (54/126), *p* < 0.01 for >50 cases and no more than 50 cases per year of endovascular intervention, 96.5% (191/198) and 73.9% (17/23), *p* < 0.01 for >10 cases and no more than 10 cases per year of kidney biopsy, respectively.

Thus, there were significant differences in the frequencies of nephrologist involvement for clinical intervention among the groups categorized by the number of volume of cases. And higher volume centers were offering significant higher frequencies of nephrologist involvement in all 4 procedures.

### Associations between the levels of nephrologist involvement and procedure volume

**Fig. 3:**
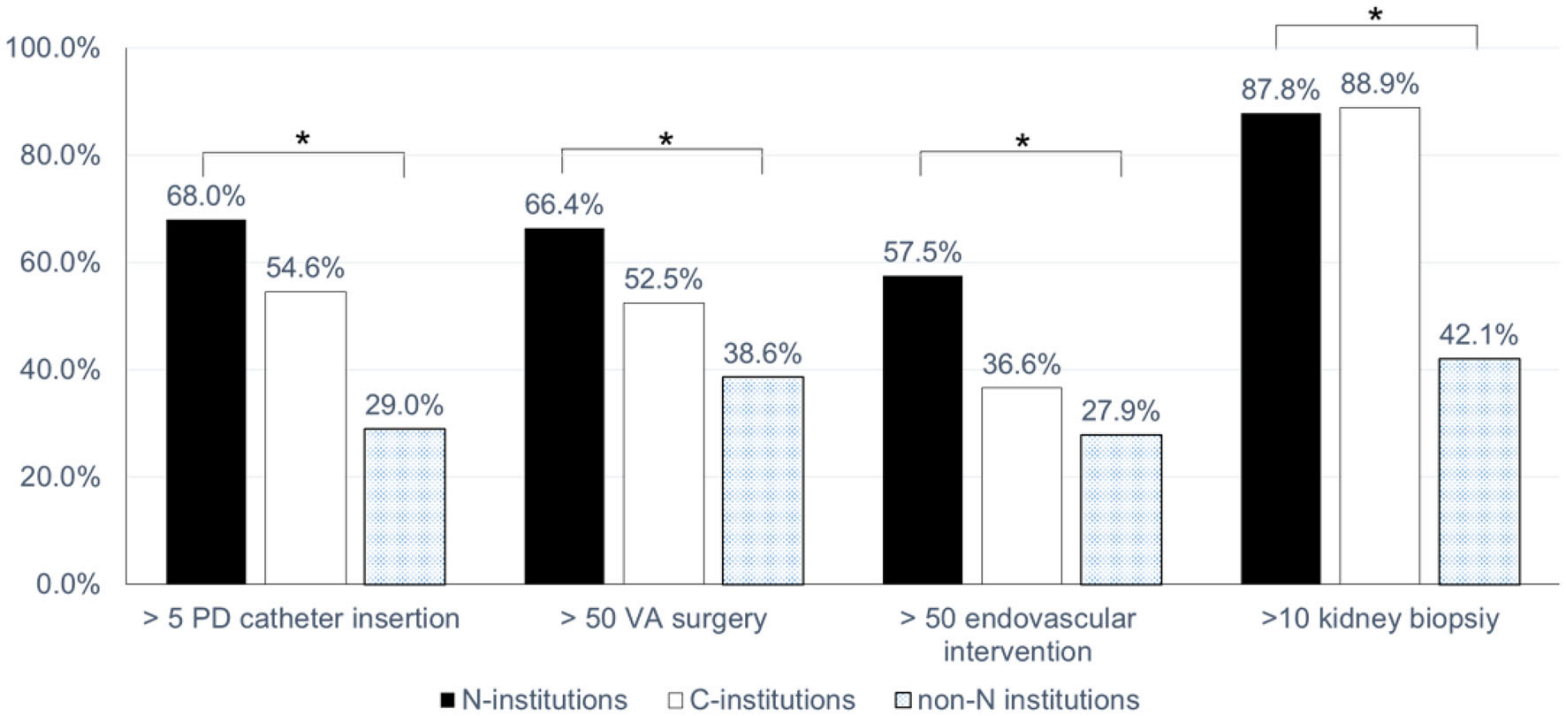
Associations between procedure manager and procedure volume. Associations between procedure manager and procedure volume were analyzed. Institutions number which were performing each procedure included in this analysis were as follows: PD (*n* = 250), VA surgery (*n* = 316), endovascular intervention (*n* = 290) and kidney biopsy (*n* = 309). Among N-institutions, C-institutions and non-N-institutions, the percentages of institutions that performed the indicated annual volumes were compared as follows: >5 PD catheter insertions [68.0% (51/75) vs 54.6% (24/44) vs 29.0% (38/131), total analyzed institutions 250], >50 VA surgery [66.4% (73/110) vs 52.5% (32/61) vs 38.6% (56/145), total analyzed institutions 316], >50 endovascular interventions [57.5% (65/113) vs 36.6% (15/41) vs 27.9% (38/136), total analyzed institutions 290], and >10 kidney biopsies [85.1% (231/263) vs 88.9% (24/27) vs 42.1% (8/19), total analyzed institutions 309]. Cochran–Armitage analyses demonstrated significant increases in procedure volume with greater management by nephrologists. Values represent the percentage of institutions which performed indicated procedure volume. *p < 0.01 for trend. *PD* peritoneal dialysis, *VA* vascular access

Next, to examine whether interventional nephrologists have the potential to increase procedure volumes as nephrologists get more deeply involved in each procedure, we analyzed associations between levels of independence from non-nephrologists and annual procedure volume. Frequencies of institutions that performed >5 PD catheter insertions, >50 VA surgery, >50 endovascular interventions and >10 kidney biopsies annually were compared among N-institutions, C-institutions and non-N-institutions (Fig. 3). Institutions which were performing each procedure were included in this analysis and each institution number was as follows: PD catheter insertion (*n* = 250), VA surgery (*n* = 316), endovascular intervention (*n* = 290) and kidney biopsy (*n* = 309).

>5 PD catheter insertions were performed in 68.0% of N-institutions (51/75), 54.6% of C-institutions (24/44) and 29.0% of non-N-institutions (38/131) among 250 institutions that were performing PD access procedures. Over 50 VA surgery were performed annually in 66.4% of N-institutions (73/110), 52.5% of C-institutions (32/61) and 38.6% of non-N-institutions (56/145) among 316 institutions that were performing VA surgery. Over 50 endovascular interventions were performed annually in 57.5% of N-institutions (65/113), 36.6% of C-institutions (15/41) and 27.9% of non-N-institutions (38/136) among 290 institutions that were performing endovascular interventions. More than 10 kidney biopsies annually were performed in 85.1% of institutions (263/309), comprising 87.8% of N-institutions (231/263), 88.9% of C-institutions (24/27) and 42.1% of non-N-institutions (8/19).

Cochran–Armitage analysis showed annual numbers of PD catheter insertions, VA surgery, endovascular interventions and kidney biopsies significantly increased in a stepwise manner from non-N-institutions to C-institutions to N-institutions as nephrologist involved more as follows: >5 PD catheter insertions/year, *n* = 250 (*p* < 0.0001); >50 VA surgery/year, *n* = 316 (*p* = 0.0055); >50 endovascular interventions/year, *n* = 290 (*p* = 0.0083); and >10 kidney biopsies/year, *n* = 309 (*p* = 0.0001). These analysis suggested procedure volume may increase with the levels of independence from non-nephrologists.

### Comparison of the annual procedure volume between institutions in which VA surgery was managed by nephrologists and non-nephrologists

**Fig. 4:**
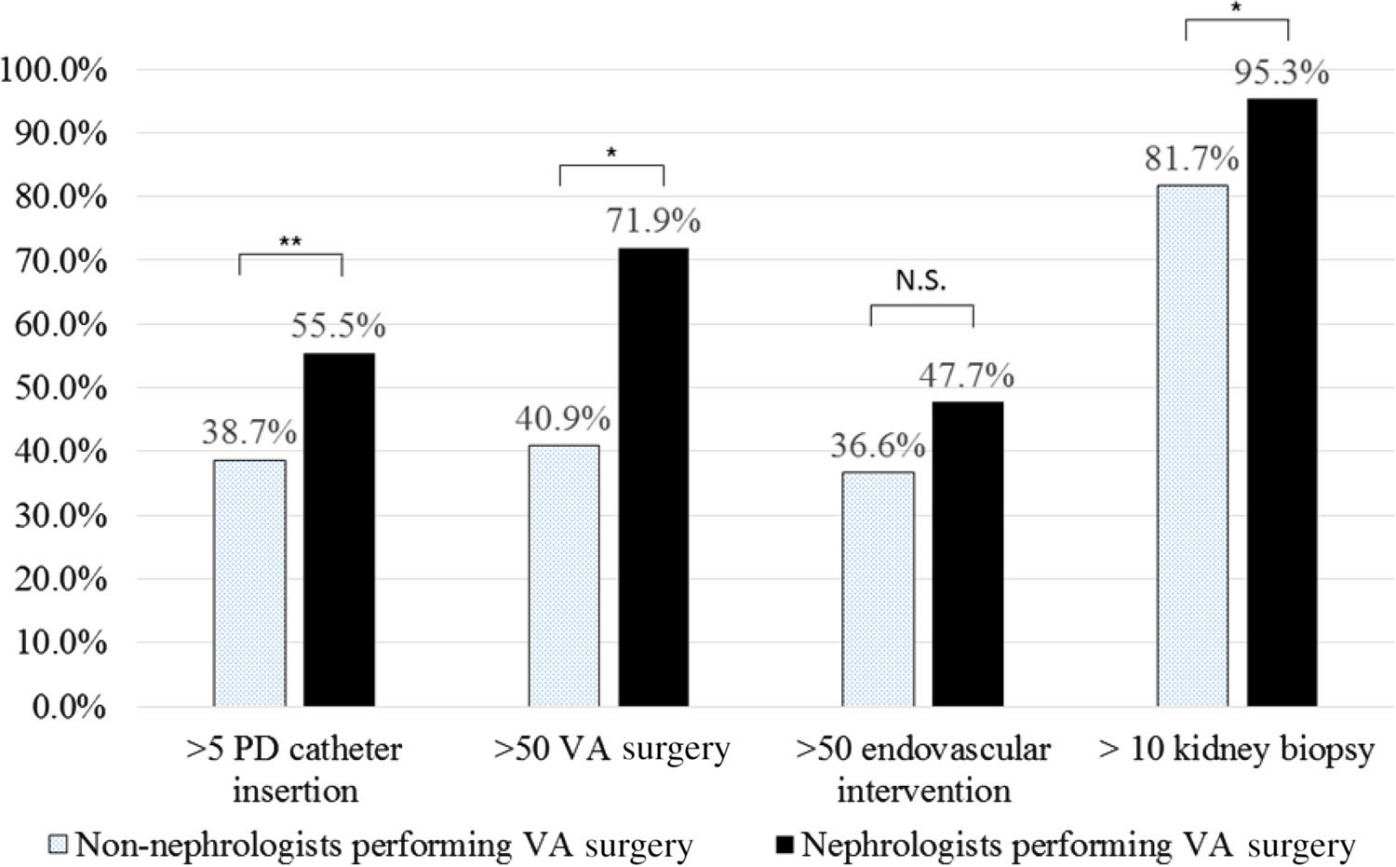
Comparison of the annual procedure volume between institutions in which VA surgery was managed by nephrologists and non-nephrologists. In this analysis, we included only 221 institutions which completed all 4 procedures in their own institutions (Fig. 1) and compared categorized procedure volume between 128 institutions in which nephrologist participated to VA surgery and 93 institutions in which VA surgery was performed by non-nephrologists alone. Each frequency of institutions performing indicated procedure volume were as follows: >5 PD catheter insertion, 55.5% (nephrologists managing institutions) vs 38.7% (non-nephrologists managing institutions), *p* = 0.0138; >50 VA surgery, 71.9% (nephrologists managing institutions) vs 40.9% (non-nephrologists managing institutions), *p* < 0.0001; >50 endovascular interventions, 47.7% (nephrologists managing institutions vs 36.6% non-nephrologists managing institutions), *p* = 0.0999; and >10 kidney biopsy, 95.3% (nephrologists managing institutions vs 81.7% non-nephrologists managing institutions), *p* = 0.0011, respectively. Values represent the percentage of institutions which performed indicated procedure volume. *p < 0.01. **p < 0.05

When nephrologists acquire a specific procedure, whether it has positive effects on the other procedure volume is worth examination. In this analysis, we included only 221 institutions which completed all 4 procedures in their own institutions (Fig. 1) and compared categorized procedure volume between 128 institutions in which nephrologist participated to VA surgery and 93 institutions in which VA surgery was performed by non-nephrologists alone (Fig. 4). Each frequency of institutions performing indicated procedure volume were as follows: for >5 PD catheter insertion, 55.5 vs 38.7%, *p* = 0.0138; for >50 VA surgery, 71.9 vs 40.9%, *p* < 0.0001; for >50 endovascular interventions, 47.7 vs 36.6%, *p* = 0.0999; and for >10 kidney biopsy, 95.3 vs 81.7%, *p* = 0.0011, respectively.

Thus, nephrologists performing VA surgery associated with procedure volume increase in both VA surgery, PD catheter insertion and kidney biopsy.

### Comparison of the annual procedure volume between institutions in which PD catheter insertion was managed by nephrologists and non-nephrologists

**Fig. 5:**
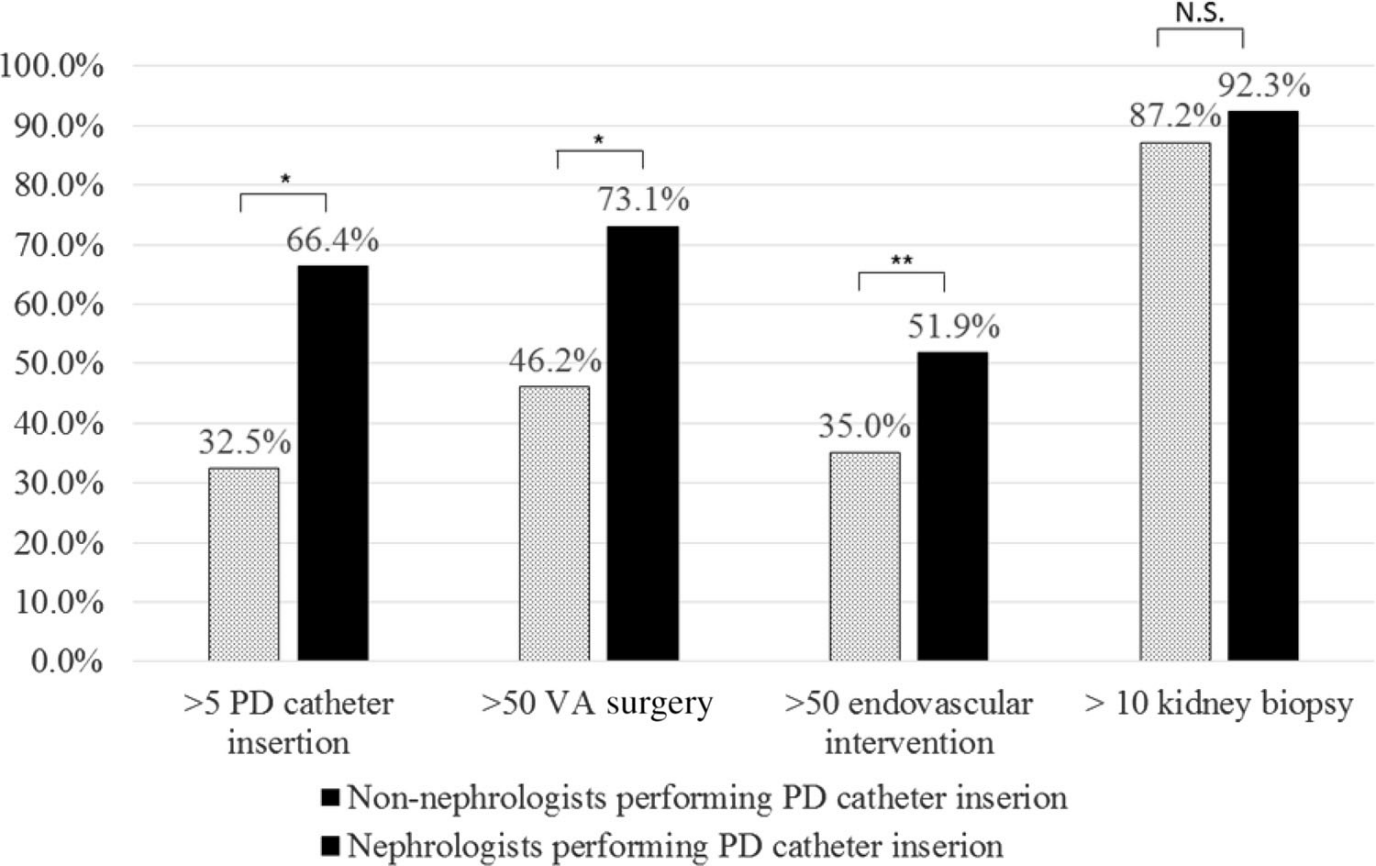
Comparison of the annual procedure volume between institutions in which PD catheter insertion was managed by nephrologists and non-nephrologists. We included only 221 institutions which performed all 4 procedures in their own institutions (Fig. 1) and compared categorized procedure volume between 117 institutions in which nephrologist participated in PD catheter insertion surgery and 104 institutions in which PD catheter insertion was performed by non-nephrologists alone (Fig. 5). Each frequency of institutions performing indicated procedure volume were as follows: >5 PD catheter insertion, 66.4% (nephrologists managing institutions) vs 32.5% (non-nephrologists managing institutions), *p* < 0.0001; >50 VA surgery, 73.1% (nephrologists managing institutions) vs 46.2% (non-nephrologists managing institutions), *p* < 0.0001; >50 endovascular interventions, 51.9% (nephrologists managing institutions) vs 35.0% (non-nephrologists managing institutions), *p* = 0.0114; and >10 kidney biopsy, 92.3% (nephrologists managing institutions) vs 87.2% (non-nephrologists managing institutions), *p* = 0.2127, respectively. Values represent the percentage of institutions which performed indicated procedure volume. *p < 0.01. **p < 0.05

As well as the above analysis, we included only 221 institutions which performed all 4 procedures in their own institutions (Fig. 1) and compared categorized procedure volume between 117 institutions in which nephrologist participated in PD catheter insertion surgery and 104 institutions in which PD catheter insertion was performed by non-nephrologists alone (Fig. 5). Each frequency of institutions performing indicated procedure volume were as follows: for >5 PD catheter insertion, 66.4 vs 32.5%, *p* < 0.01; for >50 VA surgery, 73.1 vs 46.2%, *p* < 0.01; for >50 endovascular interventions, 51.9 vs 35.0%, *p* = 0.01; and for >10 kidney biopsy, 92.3 vs 87.2%, *p* = 0.21, respectively.

Thus, when nephrologists perform PD catheter insertion, it may have positive effect on procedure volume increases in VA surgery and endovascular intervention. Additive effect of interventional nephrology on both penetration and volume increase in the other procedures was suggested in these analysis.

## Discussion

Surprisingly, main managers of all 4 procedures were nephrologists in Japan. All procedure volume increased as nephrologists become more involved. Acquisition of one specific procedure by nephrologist associated with increase in not only this specific procedure volume, but also the other procedure volume.

Thus, this first survey revealed Japanese interventional nephrology was active and Japanese nephrologists were coming in first manager of all 4 procedures in certificated Japanese nephrology training institutions and were taking the lead to perform VA surgery, endovascular intervention and PD access surgery in about a half of institutions in Japan. No other countries have been reported to offer more nephrologists’ involvement to these procedures like Japan. On the other hand, only 22.9% (75/328) of institutions could complete all 4 procedures by nephrologists (in cooperative action with non-nephrologists), indicating that majority of nephrology fellows could rarely train all 4 procedures even in Japanese certificated nephrology training institutions.

Nephrologists’ participation to VA surgery did not only associate with surgical volume increase in VA surgery, but also associated with procedure volume increase for PD catheter insertion and kidney biopsy. And nephrologists’ participation to PD catheter insertion associated with surgical volume increase in not only PD catheter insertion, but also both VA surgery and endovascular intervention. Thus, acquisition of one interventional procedure by nephrologists may enhance skill acquisition and volume increase in the other procedures. This chain reaction will activate interventional nephrology in each institution and may promote fellows to select nephrology as a carrier choice.

Japanese certificated nephrology training institutions were offering VA surgery, kidney biopsy, and endovascular intervention with higher performance rates of over 88.4% by any doctors. On the other hand, performance rate of PD access surgery by any doctors was relatively lower (76.2%) and non-nephrologists managed over half of PD access surgery, revealing many young nephrologists could not train PD access surgery even in certificated nephrology training institutions.

Present study revealed main manager of VA surgery was nephrologist, followed by cardiac surgeon, urologists and surgeon in Japan. 54.1% of nephrology training institutions were performing VA surgery by nephrologists alone (34.8%) or with the help of a non-nephrologist (19.3%), relatively high rates compared to reports from both the United States and Europe.

Arteriovenous fistula (AVF) established by nephrologists represented only 35% in Europe and under 11% in the United States [11, 12]. Beathard et al. reported nephrologist occupancy responsibility in only 25% of VA procedures and endovascular interventions in the United States, with surgeons (35%) and radiologists (30%) representing large majorities [13]. In Italy, approximately 80% of dialysis centers have performed AVF by nephrologists alone (48.8%) or with the help of a surgeon (26.4%) [14]. Thus, nephrologist-managed VA surgery is more frequently performed in Japan than the United States and Europe, but is less frequently performed than Italy.

To date, HD access surgery by nephrologists and its main effects have also been reported [12, 15–17]. VA surgery and endovascular intervention by nephrologists can improve access outcomes [18] and timely detection of malfunctions in vascular access, increases the survival of vascular access [19] and use of AVF, and decreases HD catheter utilization [20]. In these regards, frequent practice of interventional nephrology may contribute to good survival of HD patients in Japan [21].

In this survey, 76.2% institutions answered they could offer PD access surgery by any doctors. But over 10 cases/annually of PD catheter insertion were performed by only 14.3% institutions in Japan, relative fewer than 23% in USA (23%) [22]. Contrastingly, nephrologists offer PD surgery in 47.6% (119/250) institutions in Japan, relative higher than 14% in USA [1] in which non-nephrologists almost offer PD access surgery. This means non-nephrologists in USA were offering PD catheter insertion surgery more frequently than non-nephrologists in Japan.

This poor surgical volume of PD catheter insertion by non-nephrologists is a characteristic in Japan and may cause the poor penetration rate of PD in Japan [23, 24]. So, acquisition of PD catheter insertion procedure by non-nephrologists in non-N institutions may lead to increase in PD case number in Japan.

Several reports described PD catheter insertion by nephrologists and the effects on clinical nephrology, such as utilization of PD [25–28] or prevention of catheter-related infection [29]. It may be a good idea to impose obligation to participate in the PD access procedure on the non-nephrologists receiving certified member license of Society for Dialysis.

Performance rates for kidney biopsy performed by any type of doctor were 94.2% (309/328 institutions) in present study, broadly comparable to the high rate in the United States (99%) [1]. Additionally, 99.0% (306/309 institutions) of institutions performed US-guided kidney biopsy in present study, more frequent than the 42% in the United States in 2008 [1].

### Limitations

This study involved several limitations. First, participation was voluntary, with 37.8% of directors were not responding. Second, we could not analyze details about which kinds of VA surgery and endovascular intervention were performed in responding institutions. Third, no attempt was made to verify the accuracy of the responses. In this regard, other surveys conducted in this field were similar to the other survey [30, 31]. Forth, this study used a cross-sectional design so that it cannot be demonstrated that nephrologists could increase all procedure volume and acquisition of a specific procedure by nephrologists could increase the other procedure volume.

### Conclusion

This survey offers the first comprehensive picture of interventional nephrology in Japanese nephrology training institutions. Main manager of all 4 procedures was nephrologist in Japan. All procedure volume increased as nephrologists become more involved. Acquisition of one specific procedure by nephrologist associated with increase in not only this specific procedure volume, but also the other procedure volume.
